# Molecular Cloning, Characterization and Expression Analysis of the *SAMS* Gene during Adventitious Root Development in IBA-Induced Tetraploid Black Locust

**DOI:** 10.1371/journal.pone.0108709

**Published:** 2014-10-06

**Authors:** Jine Quan, Sheng Zhang, Chunxia Zhang, Sen Meng, Zhong Zhao, Xuexuan Xu

**Affiliations:** State Key Laboratory of Soil Erosion and Dryland Farming on the Loess Plateau, Northwest A&F University, Yangling, China; Tulane University Health Sciences Center, United States of America

## Abstract

S-Adenosylmethionine synthetase (SAMS) catalyzes the synthesis of S-adenosylmethionine (SAM), a precursor for ethylene and polyamine biosynthesis. Here, we report the isolation of the 1498 bp full-length cDNA sequence encoding tetraploid black locust (*Robinia pseudoacacia* L.) SAMS (*TrbSAMS*), which contains an open reading frame of 1179 bp encoding 392 amino acids. The amino acid sequence of TrbSAMS has more than 94% sequence identity to SAMSs from other plants, with a closer phylogenetic relationship to SAMSs from legumes than to SAMS from other plants. The TrbSAMS monomer consists of N-terminal, central, and C-terminal domains. Subcellular localization analysis revealed that the TrbSAMS protein localizes mainly to in the cell membrane and cytoplasm of onion epidermal cells and *Arabidopsis* mesophyll cell protoplasts. Indole-3-butyric acid (IBA)-treated cuttings showed higher levels of *TrbSAMS* transcript than untreated control cuttings during root primordium and adventitious root formation. *TrbSAMS* and its downstream genes showed differential expression in shoots, leaves, bark, and roots, with the highest expression observed in bark. IBA-treated cuttings also showed higher SAMS activity than control cuttings during root primordium and adventitious root formation. These results indicate that *TrbSAMS* might play an important role in the regulation of IBA-induced adventitious root development in tetraploid black locust cuttings.

## Introduction

S-Adenosylmethionine synthetase (SAMS) catalyzes the conversion of methionine and ATP into S-adenosylmethionine (SAM). In plants, SAM serves as a methyl-group donor during the transmethylation of lignin, DNA, RNA, and protein [1]. SAM is also a common precursor in the biosynthesis of ethylene, polyamines, and cell wall constituents [2]. In the polyamine biosynthetic pathway, S-adenosylmethionine decarboxylase (SAMDC) converts SAM to decarboxylated SAM, which polyamine oxidase (PAO) then converts to polyamines (spermine or spermidine and putrescine) [3]. Polyamines are involved in the plant rooting process, and PAO affects the formation of plant adventitious roots [4]. SAMDC and the modulation of its activity by polyamine biosynthetic inhibitors have been studied in some plants [5]. In addition, the phytohormone ethylene functions in various aspects of plant growth and development [6]. In the ethylene biosynthetic pathway, 1-aminocyclopropane-1-carboxylic acid synthase (ACS) converts SAM to 1-aminocyclopropane-1-carboxylic acid (ACC), and then ACC oxidase (ACO) converts ACC to ethylene [7]. The *ACS* gene is highly expressed during adventitious root development in *Arabidopsis thaliana* [8], [9]. The importance of SAMS is further reflected in the fact that ACS, SAMDC, and PAO have vital functional roles in the ethylene and polyamine biosynthetic pathways, respectively [2]. Genes encoding SAMS have been cloned from bacteria [10], yeasts [11], humans [12], animals [13] and plants. With regard to plants, SAMS sequences have been reported for *A. thaliana* [14], [15], *Triticum aestivum* [16], *Populus trichocarpa* [17], *Lycoris radiata* [18], *Brassica rapa* [19], *Pinus contorta* [20], Chinese cabbage (*Brassica oleracea*) [21], and mustard (*Brassica juncea*) [22], [23]. However, there are no reports of cDNA clones of SAMS in tetraploid black locust.

Black locust (*R. pseudoacacia L*.) is an attractive ornamental tree that also has various commercial uses. Homologous tetraploid black locust cultivars, generated by artificially inducing chromosome doubling in diploid locust cells, have several desirable traits. For example, due to its drought resistance and salt tolerance, t etraploid black locust is a preferred tree species for blocking wind, preventing the erosion of sand, and conserving water and soil in the northwestern Loess Plateau region. Furthermore, stem cuttings of woody plants provide a simple and economical method of vegetative propagation in the horticultural industry for rapid mass production [24], [25]. The treatment of cuttings with the auxin indole-3-butyric acid (IBA) has been reported to significantly improve the rooting rate in walnut (*Terminalia tomentosa*) [4], *P. contorta* [20], [26], apple (*Malus pumila*) [15], and *Pinus radiata* [27]. These results indicated that IBA may directly or indirectly induce root primordium formation and differentiation. Our previous study also revealed that 5.4 mM IBA significantly increases the rooting rate to approximately 80% in softwood cuttings of tetraploid black locust [24], [25], in contrast to the approximately 2% root formation that was observed in untreated control cuttings. Additionally, a *SAMS* with highly expressed during IBA-induced adventitious root development in softwood cuttings of tetraploid black locust was identified using two-dimensional electrophoresis and mass spectrometry. Pommerrenig et al. reported that *SAMS* is a key gene in the 5′-methylthioadenosine metabolic cycle, and the products of this cycle play an important role in the development of bark bundles in plants [28]. Genes related to the auxin-mediated induction of adventitious roots in forest species have been previously described [29], [30], but our understanding of the interaction between IBA and SAMS remains limited at the molecular level during adventitious root formation in *P. contorta* stem cuttings [20], [31]. For example, the interaction between IBA and *SAMS* and key downstream genes, such as *SAMDC*, *PAO*, and *ACS*, during adventitious root development in tetraploid black locust stem cuttings remains unclear.

Here, we describe the isolation and functional characterization of a full-length *SAMS* cDNA from tetraploid black locust. To investigate whether the *SAMS* gene from tetraploid black locust is related to adventitious root development, we analyzed the differential expression of *SAMS* and its downstream genes during the adventitious root development of this organ. This work aims to further explore the biological functions of *SAMS* to provide a theoretical basis for the molecular mechanisms of IBA-induced adventitious root development of softwood cuttings in tetraploid black locust.

## Materials and Methods

### Plant materials, growth conditions, and auxin-induced adventitious root development

Cuttings of tetraploid black locust (*R. pseudoacacia* L.) were collected from a 3-year-old field-grown mother stock orchard at the nursery of Northwest Agriculture and Forestry University Yangling, China. Cuttings of about 15 cm in length and 10–12 mm in diameter were collected from the sub-terminal part of shoots that were 40–50 cm in length. The basal 2.5 cm of each cutting was then dipped in cold water for the control treatment or in 5.4 mM IBA for 4 h as the auxin treatment. The cuttings of tetraploid black locust were subsequently placed on a bench in a glasshouse equipped with an automatic misting system, and a 5 cm portion of the basal part was buried in sand. The air temperature in the glasshouse was maintained at 18–28°C, with 70–90% relative humidity. During rooting, intermittent misting was supplied for 10 s at 10 min intervals before the callus appeared and for 20 s at 30 min intervals after the callus appeared. Cuttings were randomly selected from the groups treated with IBA and the controls at 0, 15, 20, 25, and 30 days after planting in a complete randomized design with 4 replicates, as described by Wang et al. [24], [25]; a total of 10 cuttings were used for one replicate. Of the four replicates, three replicates were used for the experiment, and one replicate was used for backup. These samples were taken before planting from the basal 2 cm bark of the cuttings, frozen immediately in liquid nitrogen, and stored at −80°C prior to RNA extraction.

### RNA extraction and cDNA isolation

Total RNA was extracted from the bark of soft cuttings using the Trizol reagent (Invitrogen, CA, USA) according to the manufacturer's instructions. RNA was quantified and evaluated for purity by UV spectroscopy and agarose gel electrophoresis. Prior to reverse transcription, RNA samples were treated with DNase I (Takara, Dalian, China) according to the manufacturer's manual.

Reverse transcription polymerase chain reaction (RT-PCR) was performed using a TaKaRa RNA PCR Kit (Takara, Dalian, China). Degenerate primers designed based on conserved sequences from genes encoding SAMS in other plants were used to amplify the core fragments (Table 1). PCR was performed in a 25 µl mixture containing 20 ng of template cDNA, 200 mM of each dNTP, 1.5 mM MgCl_2_, 1.0 mM of each primer, 1× PCR buffer, and 1.0 U of Taq DNA polymerase. The reaction was carried out under the following conditions: the template was denatured at 94°C for 4 min, followed by 35 cycles of 94°C for 30 s, 55°C for 30 s, and 72°C for 1 min, with a final step at 72°C for 10 min. The PCR products were separated by 1.0% (w/v) agarose gel electrophoresis, and the DNA was purified from the excised gel fragments using Agarose Gel DNA Purification Kit Ver.2.0 (Takara, Dalian, China).

**Table 1:** Primers used in the study.

Name	Sequence(5′-3′)	Purpose
TrbSAMS-F	GCACYAAGACYAACWTGGTYATGGT	Degenerate primers
TrbSAMS-R	GCTTSACCACYTCCCATGTGAAGTC	
TrbSAMS-GSP-F	GGAGTGCCTGAACCTTTATCTGT	5′RACE
TrbSAMS-GSP-R	GCCTAAGCCAAGGGCAGGTTCCATTC	3′RACE
TrbSAMS-F1	GTTATTAAAGAAGGAGGAGACTTTCC	Amplification full-length cDNA
TrbSAMS-R1	TGAGAGCAGGAGAATCACTTCACTTA	
TrbSAMS-GFP-F	CGCggatccATGGCGGAGACTTTCCTTTTTACC	SAMS-GFP subcellular localization
TrbSAMS-GFP-R	CCgagctcTTAAGCCTTCTCCCACTTGAGA	
TrbSAMS -F2	TCCTCATGGTGATGCTGGTCTC	qRT –PCR
TrbSAMS -R2	AGCAAGTCCACTGGCAACAATG	
TrbPAO-F	TGGTCACGGTCTTATGGTCAG	
TrbPAO-R	GGTACAGCAATGATAGCAGCAT	
TrbACS- F	CGGGATTTGAGATGGAGAACAG	
TrbACS- R	GTGCCTAATGGGTTTGATGGG	
TrbSAMDC - F	ACTCTGCCTCTGCTGATTCTGT	
TrbSAMDC - R	CACGGCTGCTGAACCTGTCT	
18S-F	TAGTTGGTGGAGCGATTTGTC	
18S-R	CAGAACATCTAAGGGCATCACAG	

Note: Lowercase letters indicate restriction sites.

### Amplification of the full-length *SAMS* cDNA

From the DNA fragment obtained by the above procedure, two gene-specific primers, TrbSAMS-GSP-F for 5′-RACE and TrbSAMS-GSP-R for 3′-RACE, were designed (Table 1). The 3′-cDNA ends and 5′-cDNA ends were amplified using the SMART RACE cDNA Amplification Kit according to the manufacturer's manual (Clontech, Palo Alto, CA, USA). The amplification program was as follows: denaturation at 94°C for 4 min; 5 cycles at 94°C for 30 s and 72°C for 3 min; 5 cycles at 94°C for 30 s, 70°C for 30 s, and 72°C for 3 min; 27 cycles at 94°C for 30 s, 60°C for 30 s, and 72°C for 3 min; and 10 min at 72°C. As the 5′-RACE and 3′-RACE PCR products were smeared two anchor primers, GSP1-2 for 5′-RACE and GSP2-2 for 3′-RACE, were designed for nested PCR. This approach used the smear products as a template to amplify the 5′- and 3′-cDNA ends. The PCR products were purified by gel extraction and cloned into the pMD-18T vector, and recombinant clones were sequenced by the TaKaRa Biotechnology Company (Dalian, China). By comparing and aligning internal DNA fragments from the 5′-RACE and 3′-RACE product sequences using the BioXM software (Version 2.6) package, the full-length cDNA sequence was deduced and obtained through RT-PCR amplification, which was performed with the gene-specific primers TrbSAMS-F1 and TrbSAMS-R1 (Table 1). The full-length cDNA was named TrbSAMS.

### Bioinformatic analysis

Database searches for similarity were performed in the GenBank database using the BLASTN and BLASTX algorithms. The sequences were translated to identify open reading frames using the ORF Finder in the NCBI database (http://www.ncbi.nlm.nih.gov/gorf/gorf.html). Multiple alignments of the deduced amino acid sequences were constructed using DNAMAN, and a phylogenetic tree was constructed using MEGA 5.0. The SAMS hydrophilic and hydrophobic regions were predicted using ProtScale (http://web.expasy.org/protscale/), and SAMS protein phosphorylation sites were identified using the NetPhos 2.0 Server (http://www.cbs.dtu.dk/services/NetPhos/). Additionally, the tertiary structures of SAMS proteins were analyzed using the SWISS-MODEL server (http://swissmodel.expasy.org/).

### Subcellular localization

The entire coding sequence (CDS) of *TrbSAMS* was amplified with primers incorporated *Xba*I-*Kpn*I sites at each terminus (Table 1) and ligated into the same sites of pBI221-GFP (green fluorescent protein) to produce TrbSAMS-GFP fusion protein driven by CaMV35S promoter. The PCR product was cloned into the vector pBI221-GFP to generate pBI221-TrbSAMS-GFP constructs. The resulting plasmids were confirmed by sequencing and further used for subcellular localization. Transient expression assays in onion epidermal cells were conducted using a helium biolistic device (Bio-Rad PDS-1000, Richmond, CA, USA), and a localization assay was carried out as described by Mare et al. [32]. Mesophyll protoplasts were isolated from 4-week-old *Arabidopsis* ecotype Col-0 plants and polyethylene glycol-mediated transformation assays were conducted according to the method described by Yoo et al. [33]. The transformed materials were incubated in darkness at 24°C for 16-18 h in a growth chamber. The localization of the fusion protein was observed using a confocal microscope (LSM510; Carl Zeiss, Oberkochen, Germany). GFP fluorescence, the bright field image, and the red autofluorescence of chloroplasts from protoplast expression assay were imaged simultaneously and merged together. All transient expression assays were repeated at least three times.

### Quantitative RT-PCR (qRT-PCR)

RNA was extracted from the shoots, leaves, and bark of the basal 2.5 cm of each cutting, and from the roots of adventitious root formation-phase cuttings of tetraploid black locust as described above. Reverse transcription was performed with 1 µg of RNA and the PrimeScript RT reagent Kit (Takara, Dalian, China). All of the reverse transcripts were adjusted with double-distilled water to a concentration of 150 ng/µl. Sense and antisense primers were designed using Primer Premier 5.0. RT-PCR was performed using TaKaRa SYBR Premix Ex Taq II (Perfect Real Time) on a Bio-Rad IQ5 Real-Time PCR Detection System (Bio-Rad Laboratories, Hercules, CA, USA). The volume of the qRT-PCR amplification was 25 µl, and 18S rRNA was used as the endogenous reference gene. The qRT-PCR program included a pre-denaturation step at 94°C for 2 min for fluorescence collection; amplification was then conducted for 45 cycles of 94°C for 10 s, annealing at 60°C for 15 s, and elongation at 72°C for 30 s. For the first cycle, we used a melting curve to ensure that the primers could not form dimers. Relative target gene expression was determined using the 2^−ΔΔCt^ method [13]. All reactions were run in triplicate for each sample. All data obtained from the qRT-PCR analysis were log transformed prior to data analysis using Origin (Version 8.0).

### Measurement of SAMS and ACS activity, and polyamines and ethylene contents

SAMS activity in fresh samples of the basal 2 cm of cuttings was measured using an HPLC method, as described by Lindroth et al. [20]. Thermo-BioBasic SCX color spectrum column (4.6 mm×250 mm, 5 um), and ammonium formate solution was used for the mobile phase (pH = 4.0), with other parameters set as follows: flow velocity 1 ml/min, detection wavelength 254 nm, column temperature 25°C, sample amount 10 µl. ACS activity was assayed according to the spectrophotometry method described by Boller et al. [34]. Polyamines contents were assayed by HPLC (LC-2010AHT, Shimadzu, Japan), as described by Guan et al. [35]. A Kromasil reversed-phase C18 column (250 mm×4.6 mm) was used with a mobile phase consisting of 64% methanol, a sample amount of 10 µl, a flow velocity of 0.8 ml/min, a column temperature 25°C, and a detection wavelength of 254 nm. Ethylene production was measured using gas chromatography (Trace GC Ultra, America), as described by Li et al. [36]. A 2 M stainless steel packed column was used, with a hydrogen ion flame detector, a detector temperature of 150°C, an injection port temperature of 70°C, N_2_ at 40 kPa as the carrier gas, H_2_ gas at a flow velocity of 35 ml/min, and an air flow rate of 350 ml/min. Each 1 ml sample was injected using a syringe.

## Results

### IBA induced adventitious root formation in tetraploid locust

**Figure 1:**
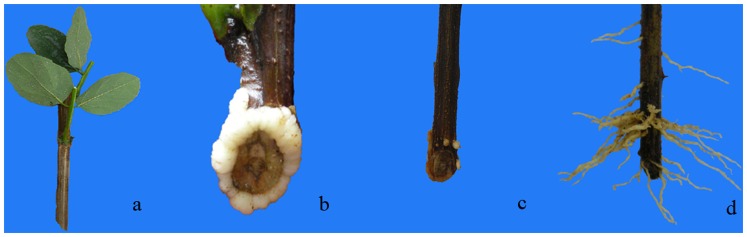
Morphological changes in tetraploid black locust cuttings undergoing adventitious root development in a sand bed. a. Softwood cuttings before cutting. b. A white callus appeared at 10 days after cutting. c. A yellow callus appeared and tiny adventitious roots emerged (root primordium) at 15 days after cutting. d. Adventitious root formation and elongation at 20 days after cutting.

**Figure 2:**
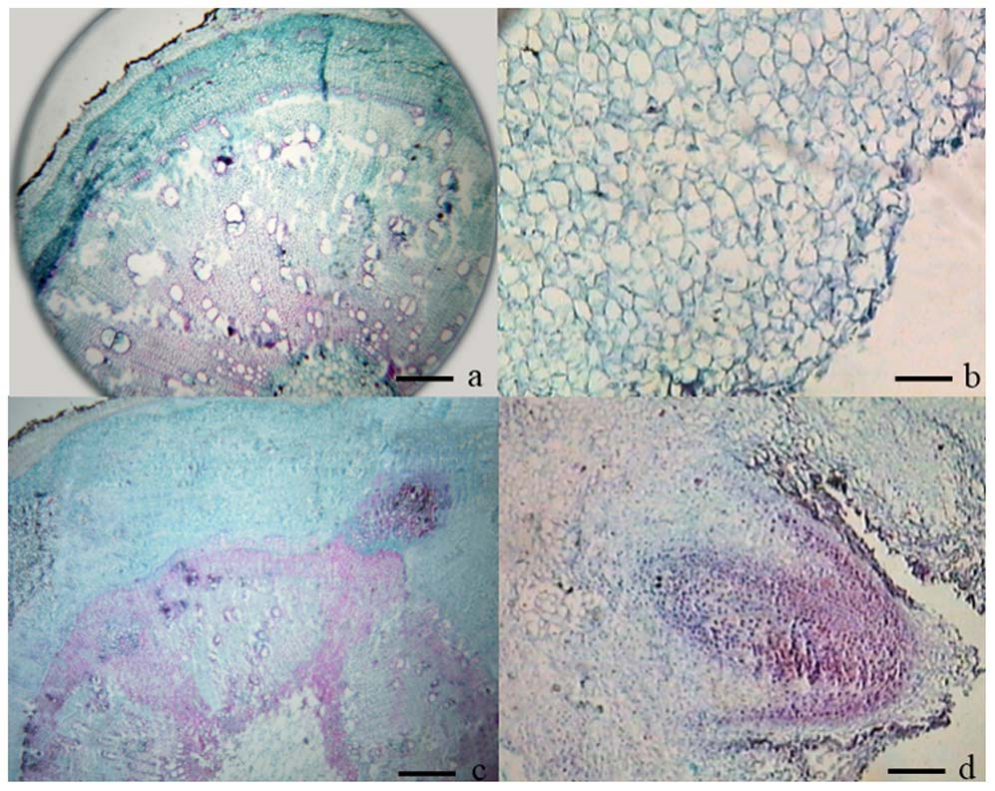
Anatomical changes in tetraploid black locust softwood cuttings undergoing IBA-induced adventitious root development. a. Cross-section of the stem before cutting. b. Parenchyma cells of callus at 10 days after cutting. c. Root primordium from the parenchyma cells at the junction between the pith rays and cortex at 15 days after cutting. d. Adventitious roots appearing at 20 days after cutting. Scale bars, 20 µm.

A system for carrying out morphological and anatomical observation of root differentiation on softwood cuttings of tetraploid black locust has been previously described [24], [25] and was further characterized in the present investigation. Nearly 80% of the softwood cuttings developed roots after treatment with an optimal concentration of IBA. Roots developed at several ranks along the entire length of the softwood cuttings (Figures 1 and 2). In the first stage, the softwood cuttings were inserted into medium beds (Figures 1a and 2a). Approximately 10 d after the IBA treatment, we detected white callus on the wound surfaces of the cuttings (Figures 1b and 2b). During the 10–15 d after the cuttings were taken, tiny adventitious root primordia formed and subsequently developed into root meristems (Figures 1c and 2c). In the last stage, adventitious roots formed and elongated (Figures 1d and 2d).

### Isolation and characterization of SAMS

As a first step in characterizing triploid locust SAMS, we obtained and sequenced a *SAMS* cDNA clone. We used 5′/3′-RACE to obtain a full-length cDNA clone, as confirmed by an analysis using DNA Star and ORF Finder. The sequence analysis confirmed the clone to be a *SAMS* gene. The full-length *SAMS* cDNA is 1498 bp with an ORF of 1179 bp, which encodes a protein of 392 amino acids; this cDNA also contains a 99 bp 5′UTR and a 220 bp 3′UTR (Figure S1). The encoded SAMS protein has a predicted molecular weight of 42.96 kDa, and a theoretical PI of 5.77. The instability index was 22.41, which indicates that the protein is stable. The full-length sequence of *TrbSAMS* was deposited in GenBank under accession number KJ940976.

### Amino acid sequence analysis and phylogenetic analysis

**Figure 3:**
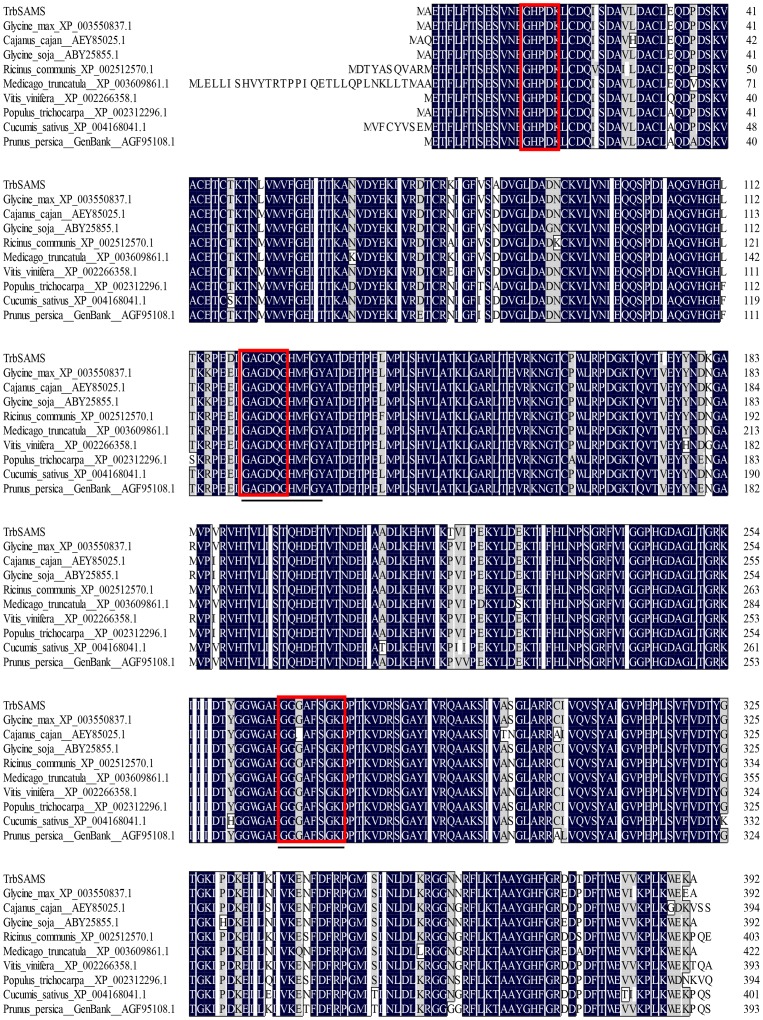
Amino acid sequence alignment of TrbSAMS with SAMS sequences from other plant species. GenBank accession numbers for nucleotide sequences: *Glycine max*, XP_003550837.1; *Cajanus cajan*, AEY85025.1; *Glycine soja*, ABY25855.1; *Ricinus communis*, XP_002512570.1; *Medicago truncatula*, XP_003609861.1; *Vitis vinifera*, XP_002266358.1; *Populus trichocarpa*, XP_002312296.1; *Cucumis sativus*, XP_004168041.1; *Prunus persica*, AGF95108.1. The same and similar amino acid residues are highlighted in black and gray respectively; the underlined sequences indicate NADP-binding sites and substrate specificity domains. Three conserved motifs are indicated in red. Two SAM synthetase signature motifs are underlined.

To further characterize TrbSAMS, we conducted a phylogenetic analysis to compare the deduced amino acid sequence of TrbSAMS with SAMS protein sequences from herbaceous and woody plants (Figure 3). The SAMS sequences showed a high degree of conservation among all the species tested. TrbSAMS showed the highest sequence identity to SAMS from *Glycine max* (97%), with SAMS amino acid sequences from *Cajanus cajan*, *Glycine soja*, *Ricinus communis*, and other plants SAMS showing 96–94% sequence identity, with some highly conserved motifs.

**Figure 4:**
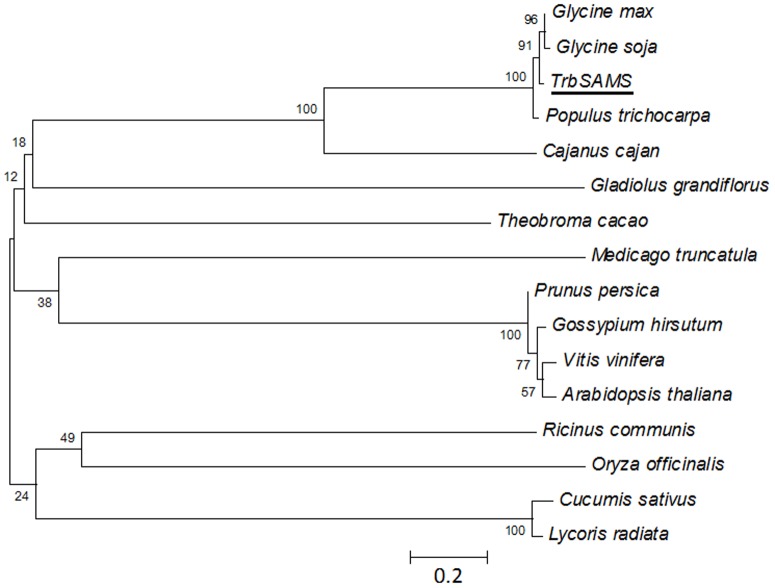
Phylogenetic tree of TrbSAMS and other plant SAMS proteins. GenBank accession number for nucleotides sequences: *Glycine max*, XP_003550837.1; *Cajanus cajan*, AEY85025.1; *Glycine soja,* ABY25855.1; *Ricinus communis*, XP_002512570.1; *Medicago truncatula*, XP_003609861.1; *Vitis vinifera*, XP_002266358.1; *Populus trichocarpa*, XP_002312296.1; *Cucumis sativus*, XP_004168041.1; *Theobroma cacao*, EOY06891.1; *Gossypium hirsutum*, ADN96174.1; *Prunus persica* AGF95108.1; *Lycoris radiata* AFC88125.1; *Arabidopsis thaliana*, NP_188365.1; *Oryza officinalis* CAJ45561.1; *Gladiolus grandiflorus*, ADM18304.1. Underlining indicates the amino acid sequence of TrbSAMS cloned in this study. The phylogenetic tree was constructed using the program MEGA 5.0. The numbers shown at internal nodes indicate the occurrence of these nodes in 1000 replicates, and the bar represents 20% sequence divergence.

To investigate the evolutionary relationship among TrbSAMS and other SAMSs, we constructed a phylogenetic tree using MEGA (Figure 4). The results revealed that SAMS proteins of plants are derived from a common ancestor and have evolved into two groups, namely dicotyledonous and monocotyledons SAMSs. TrbSAMS belongs to the dicotyledonous group and has a close relationship to SAMS from *Cajanus cajan*.

### Characterization of the deduced protein

We next examined the hydrophilic/hydrophobic nature of TrbSAMS using ProtScale software and found a maximum value of 1.867 and a minimum value of −2.267. Hydrophilic amino acid residues predominated in the peptide chain, indicating that TrbSAMS is a hydrophilic protein. We also predicted phosphorylation sites using NetPhos 2.0, which identified five Ser, seven Thr, and three Tyr phosphorylation sites in TrbSAMS. We used SOPMA to predict the secondary structure of TrbSAMS, which showed 31.63% alpha helices, 42.35% random coils, 16.84% extended strands, and 9.18% beta turns.

**Figure 5:**
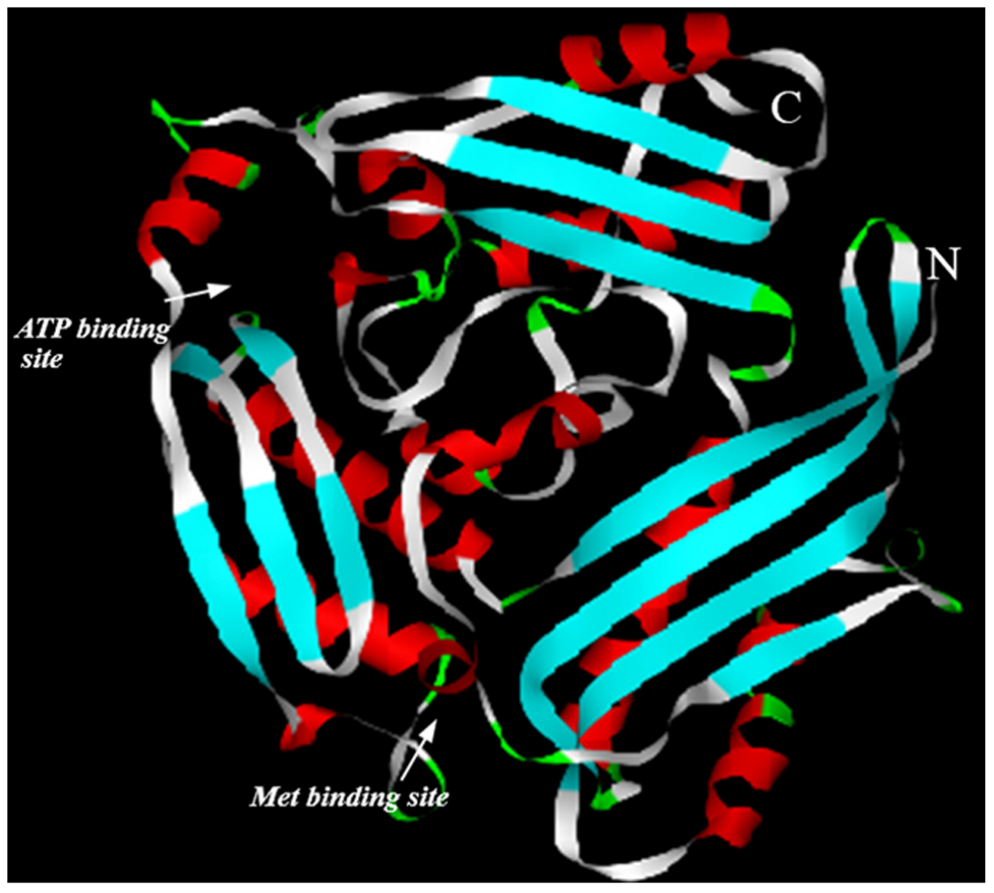
Predicated tertiary structure of TrbSAMS protein established by SWISS-MODE. The amino and carboxylic termini are labeled N and C, respectively. The entrances of substrate binding sites are marked with white arrows.

To further characterize TrbSAMS, we constructed a comparative model of its three-dimensional structure using SWISS-MODEL. As shown in Figure 5, the model of the tertiary structure of TrbSAMS indicates that the enzyme monomer consists of three domains: the N-terminal domain, the central domain and the C-terminal domain. Additionally, the model indicates two substrate binding sites, a site for ATP binding between the central and C-terminal domains, and a site for Met binding between the central and N-terminal domain. Modeling of the three-dimensional structure of TrbSAMS confirmed that it consists of three domains related to each other by a pseudo 3-fold symmetry: the N-terminal domain (5–102), the central domain (117–239), and the C-terminal domain (241–382). All these domains fold into a structure with three or four β-strands, α-helices along with an antiparallel β-strand, and α-helices on the same side as the β-strands.

### Subcellular localization of TrbSAMS

**Figure 6:**
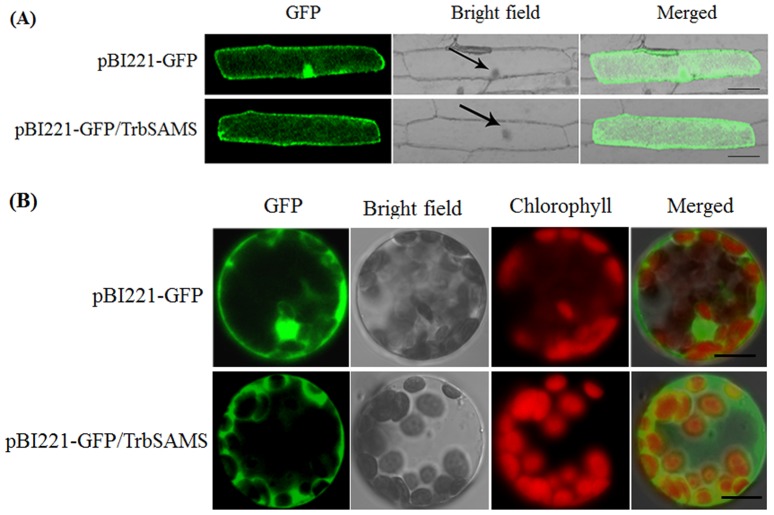
Subcellular localization of the TrbSAMS-GFP fusion protein. (A) Subcellular localization of the TrbSAMS-GFP fusion protein in onion epidermal cells. GFP fluorescence (GFP; green pseudocolor), optical photomicrographs (bright field) and an overlay of bright and GFP fluorescence illumination (merged) are shown; the arrows point to the nucleus of the cells. (B) Subcellular localization of the TrbSAMS-GFP fusion protein in *Arabidopsis* protoplasts. Images are GFP fluorescence (GFP; green pseudocolor), chlorophyll fluorescence (chlorophyll; red pseudocolor), optical photomicrographs (bright field) and merged (optical photomicrographs, chlorophyll fluorescence and GFP fluorescence). Data shown are representative of three independent experiments (n = 3). Scale bars, 200 µm.

Information on the subcellular localization of proteins can be important to elucidate the functional roles proteins play in plant cells. To examine the localization of the TrbSAMS protein, a TrbSAMS-GFP fusion construct, driven by the CaMV 35S promoter, was introduced into onion epidermal cells by particle bombardment. The results showed that TrbSAMS-GFP protein was detected mainly in the cell membrane and cytoplasm, while the control (transformation of GFP construct) was observed in the entire region of the cell (Figure 6A). To further confirm that the TrbSAMS protein is localized mainly in the cell membrane and cytoplasm, we determined the subcelluar localization of the TrbSAMS-GFP fusion protein in Arabidopsis mesophyll cell protoplasts. As shown in Figure 6B, the TrbSAMS-GFP fusion protein was preferentially localized in the cell membrane and cytoplasm and not in the nucleus. The combined results of the two experiments indicate that TrbSAMS is located mainly in the cell membrane and cytoplasm.

### Expression of *TrbSAMS* and its downstream genes at different rooting phases

**Figure 7:**
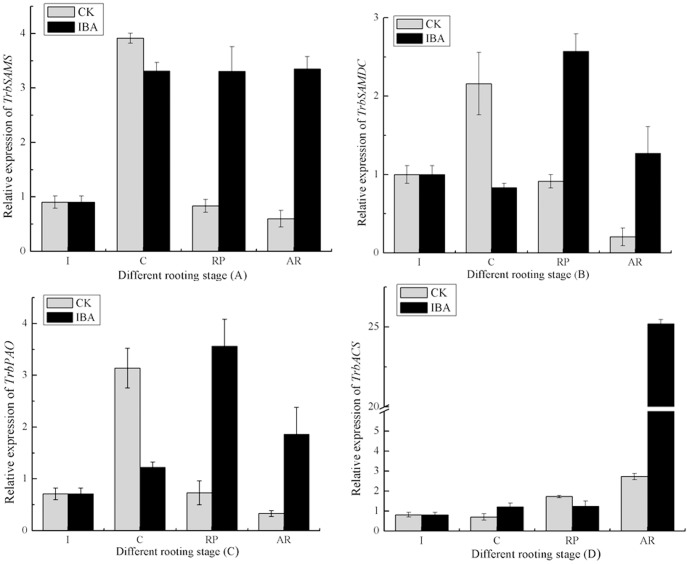
Relative expression levels of *TrbSAMS* (A), *TrbSAMDC* (B), *TrbPAO* (C), and *TrbACS* (D) during different IBA-induced and untreated rooting phases in softwood cuttings of tetraploid black locust. Initiation phase (I). Callus induction phase (C). Root primordia formation phase (RP). Adventitious root formation and elongation phase (AR). Error bars represent the standard deviation (SD) calculated from three biological replicates with IBA, CK (n = 3). CK, control treatment; IBA, indole-3-butanoic acid.

IBA-treated cuttings showed significantly higher expression (*P*≤0.01) of *TrbSAMS* compared with untreated control cuttings during the root primordia formation phase (Figure 7). *TrbSAMS* expression also remained high during the adventitious root formation phase. In contrast, untreated cuttings showed significantly higher *TrbSAMS* expression (*P*≤0.01) than IBA-treated cuttings at the callus induction phase (Figure 7A). SAM is a substrate for the synthesis of polyamines, and *SAMDC* and *PAO* are key genes for polyamine synthesis. We observed the highest expression of *TrbSAMDC* and *TrbPAO* in the root primordia formation phase in IBA-treated cuttings, and high expression in the adventitious root formation phase. We also observed lower expression in the initiation and callus induction phases. Conversely, the expression of these two genes in the root primordia formation phase and adventitious root formation phase in IBA-treated cuttings was higher than in untreated control cuttings (Figures 7B and 7C). In addition, *ACS* is a key gene in the ethylene synthesis pathway, and we observed consistently lower *TrbACS* expression from the initiation phase to the root primordia formation phase. Adventitious root formation phase IBA-treated cuttings showed significantly higher *TrbACS* (*P*≤0.01) expression than untreated control cuttings (Figure 7D). These results demonstrate that IBA might directly or indirectly regulate the expression of *TrbSAMS*, *TrbSAMDC*, *TrbPAO*, and *TrbACS* during adventitious root development in tetraploid black locust. Our results are in agreement with the expression patterns of these genes in wheat, as reported by Chai et al. [37].

### Expression of *SAMS* and its downstream genes in different tissues of tetraploid black locust

**Figure 8:**
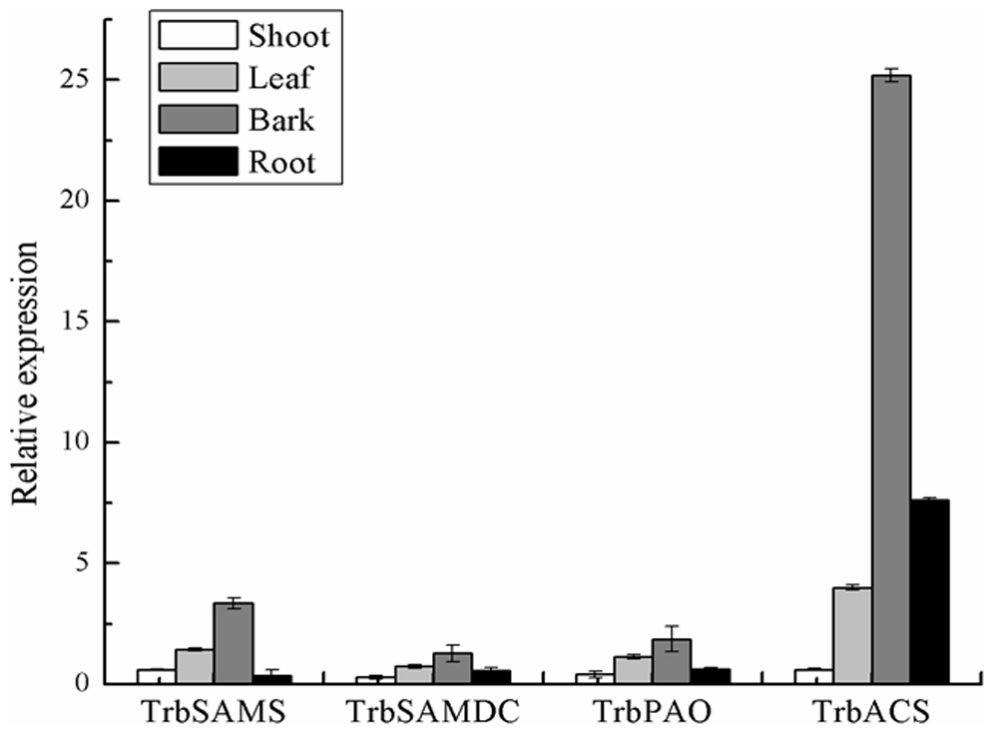
Expression of *TrbSAMS*, *TrbSAMDC*, *TrbPAO*, and *TrbACS* in the shoot, leaf, bark, and root. Error bars represent the standard deviation (SD) calculated from three biological replicates (n = 3).

We used qRT-PCR to measure the expression of *TrbSAMS* and its key downstream genes in different tissues of IBA-treated tetraploid black locust cuttings. We detected the expression of *TrbSAMS* and its key downstream genes in all organs tested, including shoots, leaves, bark, and roots, with the highest expression of these genes in the bark (Figure 8). As adventitious root formation occurs in the bark; these results suggest that *TrbSAMS* and its key downstream genes play an important role in adventitious root formation of tetraploid black locust. *TrbSAMS* expression was higher in bark than in any other tissue; the leaves and shoots showed relatively higher expression, and the roots showed low expression. With regard to the polyamine synthetic pathway, the expression of *TrbSAMDC*, *TrbPAO*, and *TrbACS* showed consistent trends during the adventitious root formation stage in different tissues of tetraploid black locust. Once again, the bark showed the highest expression of these genes, with the leaves and roots showing high expression, and the shoots showing low expression. The expression of *TrbACS* was higher in these different tissues than any other key gene (Figure 8).

### SAMS activity, ethylene and polyamines contents are increased in IBA-treated tetraploid black locust cuttings

**Figure 9:**
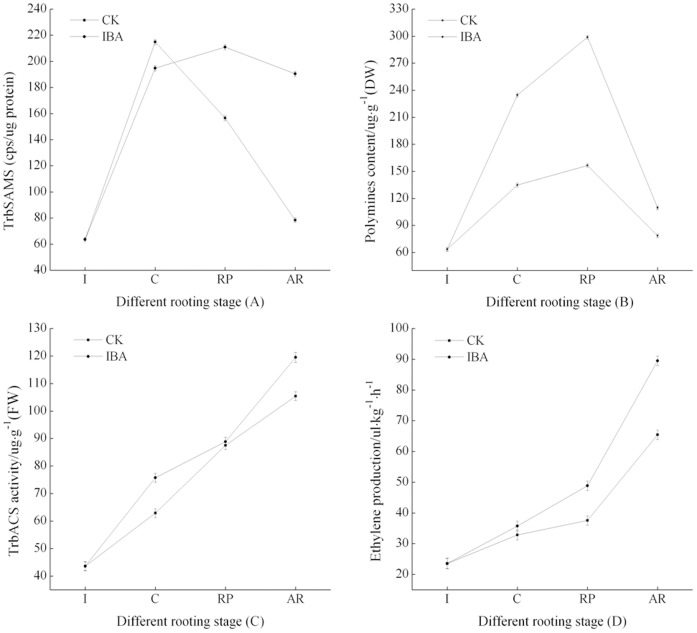
SAMS activity (A), polymines contents (B), ACS activity (C), and ethylene production (D) during the different IBA-induced and untreated rooting phase in softwood cuttings of tetraploid black locust. Initiation phase (I), callus induction phase (C), root primordia formation phase (RP), adventitious root formation and elongation phase (AR). Error bars represent the standard deviation (SD) calculated from three biological replicates with IBA, CK (n = 3). CK, control treatment; IBA, indole-3-butanoic acid.

To examine the differences between IBA-treated and untreated control cuttings, we measured SAMS activity during adventitious root development. We found that the untreated control cuttings showed the highest SAMS activity during the callus formation phase, after which SAMS activity gradually declined. The IBA-treated cuttings showed the highest SAMS activity during the root primordium formation phase, after which SAMS activity slightly declined. Additionally, the IBA-treated cuttings showed higher SAMS activity than the untreated control cuttings during the root primordium and adventitious root formation phases (Figure 9A). These results indicated that IBA treatment of cuttings might directly or indirectly enhance SAMS activity. These results are consistent with those of the Lindroth et al. [26], who examined *P. contorta* cuttings.

The untreated control and IBA-treated cuttings showed the highest polyamines contents during the root primordium formation phase, after which the polyamines contents gradually decline. Additionally, IBA treated cuttings showed higher polyamines contents than untreated control cuttings during adventitious root formation (Figure 9B). These results indicate that IBA treatment of cuttings might enhance polyamines contents. ACS activity increased gradually during adventitious root development in both the untreated control and IBA-treated cuttings. In the untreated control and IBA-treated cuttings, ethylene production likely increased gradually during adventitious root development. Additionally, the IBA-treated cuttings showed higher ACS activity and ethylene production than the untreated control cuttings during root primordium and adventitious root formation phases (Figures 9C and 9D). These results indicate that IBA treatment enhances ACS activity and thus increase ethylene contents, either directly or indirectly.

## Discussion

Previous studies of the nucleotide sequence and protein structure of SAMSs have provided evidence that SAMSs are highly conserved throughout the eukaryotic and prokaryotic kingdoms. TrbSAMS shows the highest sequence identity to *Glycine max* SAMS (97%) and the lowest sequence identity to *Ricinus communis* SAMS (94%). Although these groups tended to segregate into two divergent branches of the phylogenetic tree, they nonetheless show high amino acid sequence similarity. The results indicated that the SAMSs are relatively well conserved throughout evolution, which reflecting the importance of this enzyme for survival.

For years, auxins have been used to improve adventitious rooting for the *in vivo* or *in vitro* vegetative propagation of many herbaceous and woody species [38]. Indeed, IBA is one of the major hormone factors that affect adventitious root development in woody plant stem cuttings [15], [20], [26], [27], [31]. Our results also indicate that tetraploid black locust stem cuttings have a lower rooting rate in response to treatment with water but had a higher rooting rate in response to treatment with at 5.4 mM IBA. Recently, we used two-dimensional electrophoresis and mass spectrometry to identify a SAMS that shows high expression during IBA-induced adventitious root development of softwood cuttings in tetraploid black locust (not published). Previously, Lindroth et al. examined the effect of IBA on the expression of *SAMS* genes during the early stage of adventitious root formation, and *SAMS* that do not relate to root formation [20]. Developmental and environmental factors strictly regulate *SAMS* expression, which depends on the tissue and organ type, the stage of growth, and the response to stress [37]. SAMS is also a key enzyme in a variety of plant cell regulatory pathways.

SAMS catalyzes the synthesis of SAM, a common precursor of ethylene, and polyamine biosynthesis. Polyamines play a central role in the formation of adventitious roots [4], and polyamines are metabolic products in SAM biological pathways [38], [39]. Jarvis et al. found that auxin induced adventitious root formation in kidney beans (*Phaseolus vulgaris*) and IBA treatment increased the endogenous polyamines contents [40]. Similarly, our results showed that IBA-treated cuttings had a higher polyamines contents than untreated control cuttings during adventitious root development (Figure 9B). IBA-treated cuttings also had significantly higher SAMS activity than control cuttings during adventitious root development, consistent with the results for *P. contorta* [20]. Thus, we speculate that IBA indirectly regulates the expression of *TrbSAMS*. IBA-treated cuttings also had significantly higher *TrbSAMS* expression than control cuttings in the root primordia formation phase (*P*≤0.01, Figure 7). *SAMDC* and *PAO* are key genes in the polyamine biosynthetic pathway, and their expression levels in the root primordia and adventitious root formation phases were higher in IBA-treated cuttings than in untreated control cuttings (*P*≤0.01, Figures 7B and 7C). We therefore speculate that IBA treatment might indirectly induce *TrbSAMS*, *TrbSAMDC*, and *TrbPAO* expression during adventitious root development in softwood cuttings of tetraploid black locust.

Ethylene regulates germination, growth, development, and senescence in plants [11]; in most species, ethylene also increases adventitious root formation [41], [42], but in a few cases, inhibits or has no effect on adventitious root formation [43], [44]. Bollmark and Eliasson concluded that treatment with ACC or ethylene enhanced rooting in Norway Spruce (*Picea abies*) hypocotyl cuttings [45]. Some reports indicate a role of auxin-induced ethylene in the rooting of mung bean (*Vigna radiata*) cuttings [46]. Ragonezi et al. found that IBA can promote root formation in auxin-induced adventitious root formation in conifers, an effect that is caused by IBA increasing the content of endogenous ethylene [47]. Riov and Yang investigated mung bean cuttings, particularly hypocotyls, treated with IBA and found that these cuttings produced higher levels of ethylene and had more ACC during most of the rooting process [48]. Similarly, our results showed that IBA-treated cuttings produced more ethylene than untreated control cuttings during adventitious root development (Figure 9D). These data suggest that the stimulating effect of IBA on rooting relates closely to its induction of ACC and ethylene biosynthesis. Ethylene biosynthesis begins with the ACS enzyme forming ACC, which is then converted to ethylene by ACC oxidase [49]; and ACS is a key, rate-limiting enzyme in ethylene biosynthesis. IBA-treated cuttings showed significantly higher *TrbACS* expression than control cuttings in the adventitious root elongation phase (Figure 9D). Based on these results, we concluded that IBA affects *TrbACS* during adventitious root development in softwood cuttings of tetraploid black locust.

Previous studies have revealed that the *SAMS* gene show differential expression patterns in plants, which have been found in the vascular tissues of *Arabidopsis*, with preferential expression in roots and stems [14], [50], in rice (*Oryza sativa*) leaves [51], and in developing pea (*Pisum sativum*) ovaries [52]. Additionally, *P. contorta* has two *SAMS* genes that are differentially expressed during root development: *PcSAMS1* is preferentially expressed in roots and exhibits a specific expression pattern in the adventitious root formation phase, whereas *PcSAMS2* is expressed in roots as well as in shoots and is down-regulated during adventitious root formation [20]. Conversely, our results revealed that *TrbSAMS* is expressed of in shoots, leaves, bark, and roots, with predominant expression in the young leaves and bark of tetraploid black locust during adventitious root formation (Figure 8).

*SAMS* and the polyamines and ethylene synthesis genes *SAMDC*, *PAO*, and *ACS* showed similar expression levels in different tissues of tetraploid black locust during the adventitious root formation phase and in all organs tested, including shoots, leaves, bark, and roots, with the highest expression levels observed in bark (Figure 8). Adventitious roots form in bark, and these results indicate that *SAMS* and its downstream genes play an important role in the adventitious root formation phase of tetraploid black locust. In addition, these results indicate a possible correlation between *SAMS* expression and the expression of its downstream genes.

SAMS catalyzes the synthesis of SAM, a common precursor of ethylene, and polyamine biosynthesis. A decrease in the endogenous SAM pool via the expression of SAM hydrolase in tomato can lead to a reduction in ethylene production [53]. This further indicating that SAMS may play an important regulatory role in ethylene synthesis [22]. In addition, the over-expression or down-regulation of *SAMS* alters *ACS* expression [2]. Indeed, the ethylene biosynthesis pathway requires activated ACS through the activity of SAMS. In terms of polyamine biosynthesis, the *SAMS* gene is known to play a vital role in plant growth regulation through the synthesis of spermine or spermidine activated by SAMDC. Ge found that the over-expression or down-regulation of SAMS during *Arabidopsis* hypocotyl growth and development alters SAMDC expression [41]. To further understand the relationship between *SAMS* and its downstream genes, these genes can be examined by phenotypic and gene expression analyses of transgenic *Arabidopsis* plants.

## Conclusions

We cloned the complete ORF of *SAMS* from tetraploid black locust and analyzed the expression of this *SAMS* and its downstream genes as well as its tissue specificity, and enzyme activity in adventitious root development induced by IBA. To gain greater insight into the mechanism of SAMS function in tetraploid black locust, further research should focus on the analyses of SAMS in transgenic *Arabidopsis* plants.

## Supporting Information

Figure S1 **The cDNA and deduced amino acid sequences of TrbSAMS.** The 5′, 3′ untranslated regions are shown as lower cases. The start codon is marked with underline and the stop codon is indicated with an asterisk and an underline.
(TIF)

Materials S1 **The raw data of Figure S1.** Full-length cDNA sequence encoding tetraploid black locust (*TrbSAMS* gene) and Amino acid sequence of TrbSAMS.
(DOC)

Materials S2 **The raw data of** **Figure 3** **.** Raw data refering to amino acid sequence alignment of TrbSAMS with SAMS sequences from other plant species.
(DOC)

Materials S3 **The raw data of** **Figure 4** **.** Raw data refering to phylogenetic tree of TrbSAMS and other plant SAMS proteins.
(DOC)

Materials S4 **The raw data of** **Figure 7** **.** Raw data refering to relative expression levels of *TrbSAMS* (A), *TrbSAMDC* (B), *TrbPAO* (C), and *TrbACS* (D) during the different IBA-induced and untreated different rooting phases in softwood cuttings of tetraploid black locust. SD =  Standard Deviation, n = 3.
(DOC)

Materials S5 **The raw data of** **Figure 8** **.** Raw data refering to expression of of *TrbSAMS*, *TrbSAMDC*, *TrbPAO*, and *TrbACS* in shoot, leaf, bark, and root. SD =  Standard Deviation, n = 3.
(DOC)

Materials S6 **The raw data of** **Figure 9** **.** Raw data of SAMS activity (A), polymine content (B), ACS activity (C), and ethylene production (D) during the different IBA-induced and untreated different rooting phase of softwood cuttings in tetraploid black locust. SD =  Standard Deviation, n = 3.
(DOC)
