# Supplementary figures and images for: Molecular Cloning, Characterization and Expression Analysis of the SAMS Gene during Adventitious Root Development in IBA-Induced Tetraploid Black Locust
Source: PLoS One. 2014 Oct 6;9(10):e108709. doi: 10.1371/journal.pone.0108709 (PMC4186884; doi:10.1371/journal.pone.0108709)

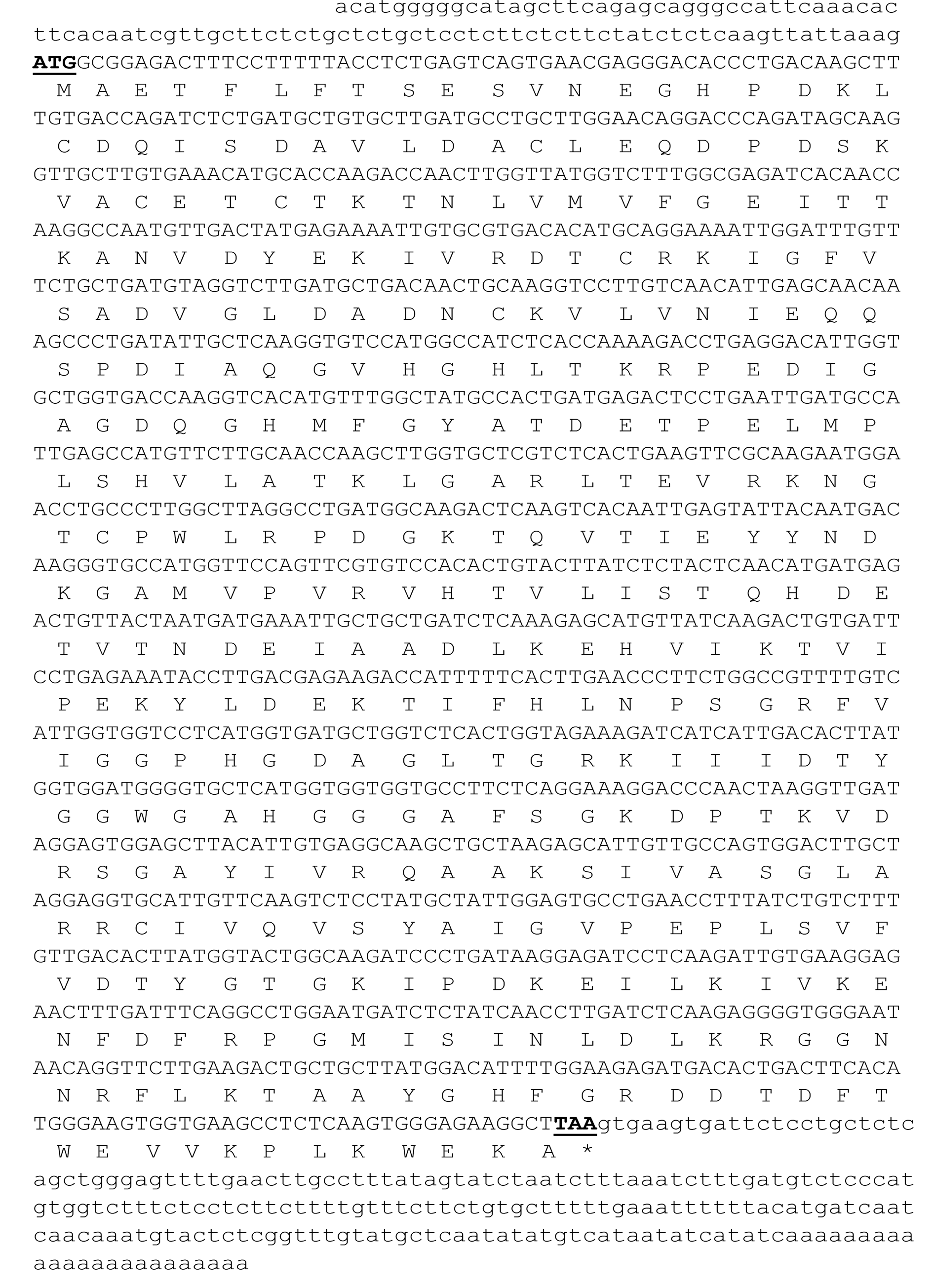

Supplement: Figure S1 — The cDNA and deduced amino acid sequences of TrbSAMS. The 5′, 3′ untranslated regions are shown as lower cases. The start codon is marked with underline and the stop codon is indicated with an asterisk and an underline. (TIF) [file pone.0108709.s001.tif]
