# Supplementary material for: Molecular Cloning, Characterization and Expression Analysis of the SAMS Gene during Adventitious Root Development in IBA-Induced Tetraploid Black Locust
Source: PLoS One. 2014 Oct 6;9(10):e108709. doi: 10.1371/journal.pone.0108709 (PMC4186884; doi:10.1371/journal.pone.0108709)
Supplement: Materials S1 — The raw data of Figure S1. Full-length cDNA sequence encoding tetraploid black locust (TrbSAMS gene) and Amino acid sequence of TrbSAMS. (DOC) [file pone.0108709.s002.doc]

**Supplementary material 2: raw data of Figure S1**

**Full-length cDNA sequence encoding tetraploid black locust (*TrbSAMS* gene).**

>SEQ1 [organism=Tetraploid Black Locust]

ATGGCGGAGACTTTCCTTTTTACCTCTGAGTCAGTGAACGAGGGACACCCTGACAAGCTTTGTGACCAGATCTCTGATGCTGTGCTTGATGCCTGCTTGGAACAGGACCCAGATAGCAAGGTTGCTTGTGAAACATGCACCAAGACCAACTTGGTTATGGTCTTTGGCGAGATCACAACCAAGGCCAATGTTGACTATGAGAAAATTGTGCGTGACACATGCAGGAAAATTGGATTTGTTTCTGCTGATGTAGGTCTTGATGCTGACAACTGCAAGGTCCTTGTCAACATTGAGCAACAAAGCCCTGATATTGCTCAAGGTGTCCATGGCCATCTCACCAAAAGACCTGAGGACATTGGTGCTGGTGACCAAGGTCACATGTTTGGCTATGCCACTGATGAGACTCCTGAATTGATGCCATTGAGCCATGTTCTTGCAACCAAGCTTGGTGCTCGTCTCACTGAAGTTCGCAAGAATGGAACCTGCCCTTGGCTTAGGCCTGATGGCAAGACTCAAGTCACAATTGAGTATTACAATGACAAGGGTGCCATGGTTCCAGTTCGTGTCCACACTGTACTTATCTCTACTCAACATGATGAGACTGTTACTAATGATGAAATTGCTGCTGATCTCAAAGAGCATGTTATCAAGACTGTGATTCCTGAGAAATACCTTGACGAGAAGACCATTTTTCACTTGAACCCTTCTGGCCGTTTTGTCATTGGTGGTCCTCATGGTGATGCTGGTCTCACTGGTAGAAAGATCATCATTGACACTTATGGTGGATGGGGTGCTCATGGTGGTGGTGCCTTCTCAGGAAAGGACCCAACTAAGGTTGATAGGAGTGGAGCTTACATTGTGAGGCAAGCTGCTAAGAGCATTGTTGCCAGTGGACTTGCTAGGAGGTGCATTGTTCAAGTCTCCTATGCTATTGGAGTGCCTGAACCTTTATCTGTCTTTGTTGACACTTATGGTACTGGCAAGATCCCTGATAAGGAGATCCTCAAGATTGTGAAGGAGAACTTTGATTTCAGGCCTGGAATGATCTCTATCAACCTTGATCTCAAGAGGGGTGGGAATAACAGGTTCTTGAAGACTGCTGCTTATGGACATTTTGGAAGAGATGACACTGACTTCACATGGGAAGTGGTGAAGCCTCTCAAGTGGGAGAAGGCTTAA

**Amino acid sequence of TrbSAMS**

>SEQ1 [gene=SAMS] [protein=S-adenosylmethionine-synthetase] Tetraploid Black Locust

MAETFLFTSESVNEGHPDKLCDQISDAVLDACLEQDPDSKVACETCTKTNLVMVFGEITTKANVDYEKIVRDTCRKIGFVSADVGLDADNCKVLVNIEQQSPDIAQGVHGHLTKRPEDIGAGDQGHMFGYATDETPELMPLSHVLATKLGARLTEVRKNGTCPWLRPDGKTQVTIEYYNDKGAMVPVRVHTVLISTQHDETVTNDEIAADLKEHVIKTVIPEKYLDEKTIFHLNPSGRFVIGGPHGDAGLTGRKIIIDTYGGWGAHGGGAFSGKDPTKVDRSGAYIVRQAAKSIVASGLARRCIVQVSYAIGVPEPLSVFVDTYGTGKIPDKEILKIVKENFDFRPGMISINLDLKRGGNNRFLKTAAYGHFGRDDTDFTWEVVKPLKWEKA
