# Supplementary material for: Molecular Cloning, Characterization and Expression Analysis of the SAMS Gene during Adventitious Root Development in IBA-Induced Tetraploid Black Locust
Source: PLoS One. 2014 Oct 6;9(10):e108709. doi: 10.1371/journal.pone.0108709 (PMC4186884; doi:10.1371/journal.pone.0108709)
Supplement: Materials S2 — The raw data of Figure 3 . Raw data refering to amino acid sequence alignment of TrbSAMS with SAMS sequences from other plant species. (DOC) [file pone.0108709.s003.doc]

**Supplementary material 3: raw data of Figure 3**

**Raw data refering to amino acid sequence alignment of TrbSAMS with SAMS sequences from other plant species.**

> *TrbSAMS* KJ940976

MAETFLFTSESVNEGHPDKLCDQISDAVLDACLEQDPDSKVACETCTKTNLVMVFGEITTKANVDYEKIVRDTCRKIGFVSADVGLDADNCKVLVNIEQQSPDIAQGVHGHLTKRPEDIGAGDQGHMFGYATDETPELMPLSHVLATKLGARLTEVRKNGTCPWLRPDGKTQVTIEYYNDKGAMVPVRVHTVLISTQHDETVTNDEIAADLKEHVIKTVIPEKYLDEKTIFHLNPSGRFVIGGPHGDAGLTGRKIIIDTYGGWGAHGGGAFSGKDPTKVDRSGAYIVRQAAKSIVASGLARRCIVQVSYAIGVPEPLSVFVDTYGTGKIPDKEILKIVKENFDFRPGMISINLDLKRGGNNRFLKTAAYGHFGRDDTDFTWEVVKPLKWEKA

> *Glycine max* XP_003550837.1

MAETFLFTSESVNEGHPDKLCDQISDAVLDACLEQDPDSKVACETCTKTNLVMVFGEITTKANVDYEKIVRDTCRNIGFVSNDVGLDADNCKVLVNIEQQSPDIAQGVHGHLTKKPEEIGAGDQGHMFGYATDETPELMPLSHVLATKLGARLTEVRKNGTCPWLRPDGKTQVTVEYYNDNGARVPVRVHTVLISTQHDETVTNDEIAADLKEHVIKPVIPEKYLDEKTIFHLNPSGRFVIGGPHGDAGLTGRKIIIDTYGGWGAHGGGAFSGKDPTKVDRSGAYIVRQAAKSIVASGLARRCIVQVSYAIGVPEPLSVFVDTYGTGKIPDKEILNIVKENFDFRPGMISINLDLKRGGNNRFLKTAAYGHFGREDPDFTWEVIKPLKWEEA

> *Cajanus cajan* AEY85025.1

MAQETFLFTSESVNEGHPDKLCDQISDAVHDACLEQDPDSKVACETCTKTNMVMVFGEITTKANVDYEKIVRDTCRNIGFVSDDVGLDADNCKVLVNIEQQSPDIAQGVHGHLTKRPEEIGAGDQGHMFGYATDETPELMPLSHVLATKLGARLTEVRKNGTCPWLRPDGKTQVTVEYYNDKGAMVPIRVHTVLISTQHDETVTNDEIAADLKEHVIKPVIPEKYLDEKTIFHLNPSGRFVIGGPHGDAGLTGRKIIIDTYGGWGAHGGAFSGKDPTKVDRSGAYIVRQAAKSIVTNGLARRAIVQVSYAIGVPEPLSVFVDTYGTGKIPDKEILSIVKENFDFRPGMISINLDLKRGGNGRFLKTAAYGHFGRDDPDFTWEVVKPLKGDKVSS

> *Glycine soja* ABY25855.1

MAETFLFTSESVNEGHPDKLCDQISDAVLDACLEQDPDSKVACETCTKTNLVMVFGEITTKANVDYEKIVRDTCRNIGFVSNDVGLDAGNCKVLVNIEQQSPDIAQGVHGHLTKKPEEIGAGDQGHMFGYATDETPELMPLSHVLATKLGARLTEVRKNGTCPWLRPDGKTQVTVEYYNDNGARVPIRVHTVLISTQHDETVTNDEIAADLKEHVIKPVIPEKYLDEKTIFHLNPSGRFVIGGPHGDAGLTGRKIIIDTYGGWGAHGGGAFSGKDPTKVDRSGAYIVRQAAKSIVASGLARRCIVQVSYAIGVPEPLSVFVDTYGTGKIHDKEILNIVKENFDFRPGMISINLDLKRGGNNRFLKTAAYGHFGREDPDFTWEVVKPLKWEKA

> *Ricinus communis* XP_002512570.1

MDTYASQVARMETFLFTSESVNEGHPDKLCDQVSDAILDACLEQDPDSKVACETCTKTNMVMVFGEITTKANVDYEKIVRDTCRAIGFVSDDVGLDADKCKVLVNIEQQSPDIAQGVHGHLTKRPEEIGAGDQGHMFGYATDETPEFMPLSHVLATKLGARLTEVRKNGTCPWLRPDGKTQVTVEYYNDNGAMVPVRVHTVLISTQHDETVTNDEIAADLKEHVIKPVIPEKYLDEKTIFHLNPSGRFVIGGPHGDAGLTGRKIIIDTYGGWGAHGGGAFSGKDPTKVDRSGAYIVRQAAKSIVANGLARRCIVQVSYAIGVPEPLSVFVDTYGTGKIPDKEILKIVKESFDFRPGMISINLDLKRGGNGRFLKTAAYGHFGRDDSDFTWEVVKPLKWEKPQE

> *Medicago truncatula* XP_003609861.1

MLELLISHVYTRTPPIQETLLQPLNKLLTMAAETFLFTSESVNEGHPDKLCDQISDAVLDACLEQDVDSKVACETCTKTNLVMVFGEITTKAKVDYEKIVRDTCRKIGFVSDDVGLDADNCKVLVNIEQQSPDIAQGVHGHLTKRPEEIGAGDQGHMFGYATDETPELMPLSHVLATKLGARLTEVRKNGTCPWLRPDGKTQVTVEYYNDNGAMVPVRVHTVLISTQHDETVTNDEIAADLKEHVIKPVIPDKYLDSKTIFHLNPSGRFVIGGPHGDAGLTGRKIIIDTYGGWGAHGGGAFSGKDPTKVDRSGAYIVRQAAKSIVASGLARRCIVQVSYAIGVPEPLSVFVDTYGTGKIPDKEILNIVKQNFDFRPGMISINLDLLRGGNGRFLKTAAYGHFGREDADFTWEVVKPLKWEKA

> *Vitis vinifera* XP_002266358.1

METFLFTSESVNEGHPDKLCDQISDAVLDACLQQDPDSKVACETCTKTNMVMVFGEITTKANVDYEKIVRDTCREIGFVSDDVGLDADNCKVLVNIEQQSPDIAQGVHGHLTKRPEEIGAGDQGHMFGYATDETPELMPLSHVLATKLGARLTEVRKNGTCPWLRPDGKTQVTVEYHNDGGARVPIRVHTVLISTQHDETVTNDEIAADLKEHVIKPVIPEKYLDEKTIFHLNPSGRFVIGGPHGDAGLTGRKIIIDTYGGWGAHGGGAFSGKDPTKVDRSGAYIVRQAAKSIVANGLARRCIVQVSYAIGVPEPLSVFVDTYGTGKIPDREILKIVKENFDFRPGMISINLDLKRGGNGRFLKTAAYGHFGRDDPDFTWEVVKPLKWEKTQA

> *Populus trichocarpa* XP_002312296.1

MAETFLFTSESVNEGHPDKLCDQISDAVLDACLAQDPDSKVACETCTKTNMVMVFGEITTKADVDYEKIVRDTCRNIGFTSADVGLDADNCKVLVNIEQQSPDIAQGVHGHFSKRPEEIGAGDQGHMFGYATDETPELMPLSHVLATKLGARLTEVRKNGTCAWLRPDGKTQVTVEYYNENGAMVPIRVHTVLISTQHDETVTNDEIAADLKEHVIKPVIPEKYLDEKTIFHLNPSGRFVIGGPHGDAGLTGRKIIIDTYGGWGAHGGGAFSGKDPTKVDRSGAYIVRQAAKSIVASGLARRCIVQVSYAIGVPEPLSVFVDTYGTGKIPDKEILQIVKESFDFRPGMISINLDLKRGGNSRFLKTAAYGHFGRDDPDFTWEVVKPLKWDNKVQA

> *Cucumis sativus* XP_004168041.1

MVFCYVSEMETFLFTSESVNEGHPDKLCDQISDAVLDACLAQDPDSKVACETCSKTNMVMVFGEITTKANVDYEKIVRDTCRNIGFISDDVGLDADNCKVLVNIEQQSPDIAQGVHGHFTKRPEEIGAGDQGHMFGYATDETPELMPLSHVLATKLGARLTEVRKNGTCPWLRPDGKTQVTVEYYNDNGAMVPVRVHTVLISTQHDETVTNDEIATDLKEHVIKPIIPEKYLDEKTIFHLNPSGRFVIGGPHGDAGLTGRKIIIDTHGGWGAHGGGAFSGKDPTKVDRSGAYIVRQAAKSIVASGLARRCIVQVSYAIGVPEPLSVFVDTYKTGKIPDKEILEIVKENFDFRPGMITINLDLKRGGNGRFLKTAAYGHFGRDDPDFTWETIKPLKWEKPQS

> *Prunus persica* AGF95108.1

METFLFTSESVNEGHPDKLCDQISDAVLDACLAQDADSKVACETCTKTNMVMVFGEITTKANVDYEKIVRETCRNIGFISDDVGLDADNCKVLVNIEQQSPDIAQGVHGHFTKRPEEIGAGDQGHMFGYATDETPELMPLSHVLATKLGARLTEVRKNGTCPWLRPDGKTQVTVEYYNENGAMVPVRVHTVLISTQHDETVTNDEIAADLKEHVIKPVVPEKYLDEKTIFHLNPSGRFVIGGPHGDAGLTGRKIIIDTYGGWGAHGGGAFSGKDPTKVDRSGAYIVRQAAKSIVANGLARRALVQVSYAIGVPEPLSVFVDTYGTGKIPDKEILKIVKETFDFRPGMITINLDLKRGGGGRFLKTAAYGHFGRDDPDFTWEVVKPLKWEKPQS
