# Supplementary material for: Molecular Cloning, Characterization and Expression Analysis of the SAMS Gene during Adventitious Root Development in IBA-Induced Tetraploid Black Locust
Source: PLoS One. 2014 Oct 6;9(10):e108709. doi: 10.1371/journal.pone.0108709 (PMC4186884; doi:10.1371/journal.pone.0108709)
Supplement: Materials S3 — The raw data of Figure 4 . Raw data refering to phylogenetic tree of TrbSAMS and other plant SAMS proteins. (DOC) [file pone.0108709.s004.doc]

**Supplementary material 4: raw data of Figure 4**

**Raw data refering to phylogenetic tree of TrbSAMS and other plant SAMS proteins.**

> *TrbSAMS* KJ940976

MAETFLFTSESVNEGHPDKLCDQISDAVLDACLEQDPDSKVACETCTKTNLVMVFGEITTKANVDYEKIVRDTCRKIGFVSADVGLDADNCKVLVNIEQQSPDIAQGVHGHLTKRPEDIGAGDQGHMFGYATDETPELMPLSHVLATKLGARLTEVRKNGTCPWLRPDGKTQVTIEYYNDKGAMVPVRVHTVLISTQHDETVTNDEIAADLKEHVIKTVIPEKYLDEKTIFHLNPSGRFVIGGPHGDAGLTGRKIIIDTYGGWGAHGGGAFSGKDPTKVDRSGAYIVRQAAKSIVASGLARRCIVQVSYAIGVPEPLSVFVDTYGTGKIPDKEILKIVKENFDFRPGMISINLDLKRGGNNRFLKTAAYGHFGRDDTDFTWEVVKPLKWEKA

> *Glycine max* XP_003550837.1

MAETFLFTSESVNEGHPDKLCDQISDAVLDACLEQDPDSKVACETCTKTNLVMVFGEITTKANVDYEKIVRDTCRNIGFVSNDVGLDADNCKVLVNIEQQSPDIAQGVHGHLTKKPEEIGAGDQGHMFGYATDETPELMPLSHVLATKLGARLTEVRKNGTCPWLRPDGKTQVTVEYYNDNGARVPVRVHTVLISTQHDETVTNDEIAADLKEHVIKPVIPEKYLDEKTIFHLNPSGRFVIGGPHGDAGLTGRKIIIDTYGGWGAHGGGAFSGKDPTKVDRSGAYIVRQAAKSIVASGLARRCIVQVSYAIGVPEPLSVFVDTYGTGKIPDKEILNIVKENFDFRPGMISINLDLKRGGNNRFLKTAAYGHFGREDPDFTWEVIKPLKWEEA

> *Cajanus cajan* AEY85025.1

MAQETFLFTSESVNEGHPDKLCDQISDAVHDACLEQDPDSKVACETCTKTNMVMVFGEITTKANVDYEKIVRDTCRNIGFVSDDVGLDADNCKVLVNIEQQSPDIAQGVHGHLTKRPEEIGAGDQGHMFGYATDETPELMPLSHVLATKLGARLTEVRKNGTCPWLRPDGKTQVTVEYYNDKGAMVPIRVHTVLISTQHDETVTNDEIAADLKEHVIKPVIPEKYLDEKTIFHLNPSGRFVIGGPHGDAGLTGRKIIIDTYGGWGAHGGAFSGKDPTKVDRSGAYIVRQAAKSIVTNGLARRAIVQVSYAIGVPEPLSVFVDTYGTGKIPDKEILSIVKENFDFRPGMISINLDLKRGGNGRFLKTAAYGHFGRDDPDFTWEVVKPLKGDKVSS

> *Glycine soja* ABY25855.1

MAETFLFTSESVNEGHPDKLCDQISDAVLDACLEQDPDSKVACETCTKTNLVMVFGEITTKANVDYEKIVRDTCRNIGFVSNDVGLDAGNCKVLVNIEQQSPDIAQGVHGHLTKKPEEIGAGDQGHMFGYATDETPELMPLSHVLATKLGARLTEVRKNGTCPWLRPDGKTQVTVEYYNDNGARVPIRVHTVLISTQHDETVTNDEIAADLKEHVIKPVIPEKYLDEKTIFHLNPSGRFVIGGPHGDAGLTGRKIIIDTYGGWGAHGGGAFSGKDPTKVDRSGAYIVRQAAKSIVASGLARRCIVQVSYAIGVPEPLSVFVDTYGTGKIHDKEILNIVKENFDFRPGMISINLDLKRGGNNRFLKTAAYGHFGREDPDFTWEVVKPLKWEKA

> *Ricinus communis* XP_002512570.1

MDTYASQVARMETFLFTSESVNEGHPDKLCDQVSDAILDACLEQDPDSKVACETCTKTNMVMVFGEITTKANVDYEKIVRDTCRAIGFVSDDVGLDADKCKVLVNIEQQSPDIAQGVHGHLTKRPEEIGAGDQGHMFGYATDETPEFMPLSHVLATKLGARLTEVRKNGTCPWLRPDGKTQVTVEYYNDNGAMVPVRVHTVLISTQHDETVTNDEIAADLKEHVIKPVIPEKYLDEKTIFHLNPSGRFVIGGPHGDAGLTGRKIIIDTYGGWGAHGGGAFSGKDPTKVDRSGAYIVRQAAKSIVANGLARRCIVQVSYAIGVPEPLSVFVDTYGTGKIPDKEILKIVKESFDFRPGMISINLDLKRGGNGRFLKTAAYGHFGRDDSDFTWEVVKPLKWEKPQE

>*Medicago truncatula* XP_003609861.1

MLELLISHVYTRTPPIQETLLQPLNKLLTMAAETFLFTSESVNEGHPDKLCDQISDAVLDACLEQDVDSKVACETCTKTNLVMVFGEITTKAKVDYEKIVRDTCRKIGFVSDDVGLDADNCKVLVNIEQQSPDIAQGVHGHLTKRPEEIGAGDQGHMFGYATDETPELMPLSHVLATKLGARLTEVRKNGTCPWLRPDGKTQVTVEYYNDNGAMVPVRVHTVLISTQHDETVTNDEIAADLKEHVIKPVIPDKYLDSKTIFHLNPSGRFVIGGPHGDAGLTGRKIIIDTYGGWGAHGGGAFSGKDPTKVDRSGAYIVRQAAKSIVASGLARRCIVQVSYAIGVPEPLSVFVDTYGTGKIPDKEILNIVKQNFDFRPGMISINLDLLRGGNGRFLKTAAYGHFGREDADFTWEVVKPLKWEKA

> *Vitis vinifera* XP_002266358.1

METFLFTSESVNEGHPDKLCDQISDAVLDACLQQDPDSKVACETCTKTNMVMVFGEITTKANVDYEKIVRDTCREIGFVSDDVGLDADNCKVLVNIEQQSPDIAQGVHGHLTKRPEEIGAGDQGHMFGYATDETPELMPLSHVLATKLGARLTEVRKNGTCPWLRPDGKTQVTVEYHNDGGARVPIRVHTVLISTQHDETVTNDEIAADLKEHVIKPVIPEKYLDEKTIFHLNPSGRFVIGGPHGDAGLTGRKIIIDTYGGWGAHGGGAFSGKDPTKVDRSGAYIVRQAAKSIVANGLARRCIVQVSYAIGVPEPLSVFVDTYGTGKIPDREILKIVKENFDFRPGMISINLDLKRGGNGRFLKTAAYGHFGRDDPDFTWEVVKPLKWEKTQA

> *Populus trichocarpa* XP_002312296.1

MAETFLFTSESVNEGHPDKLCDQISDAVLDACLAQDPDSKVACETCTKTNMVMVFGEITTKADVDYEKIVRDTCRNIGFTSADVGLDADNCKVLVNIEQQSPDIAQGVHGHFSKRPEEIGAGDQGHMFGYATDETPELMPLSHVLATKLGARLTEVRKNGTCAWLRPDGKTQVTVEYYNENGAMVPIRVHTVLISTQHDETVTNDEIAADLKEHVIKPVIPEKYLDEKTIFHLNPSGRFVIGGPHGDAGLTGRKIIIDTYGGWGAHGGGAFSGKDPTKVDRSGAYIVRQAAKSIVASGLARRCIVQVSYAIGVPEPLSVFVDTYGTGKIPDKEILQIVKESFDFRPGMISINLDLKRGGNSRFLKTAAYGHFGRDDPDFTWEVVKPLKWDNKVQA

> *Cucumis sativus* XP_004168041.1

MVFCYVSEMETFLFTSESVNEGHPDKLCDQISDAVLDACLAQDPDSKVACETCSKTNMVMVFGEITTKANVDYEKIVRDTCRNIGFISDDVGLDADNCKVLVNIEQQSPDIAQGVHGHFTKRPEEIGAGDQGHMFGYATDETPELMPLSHVLATKLGARLTEVRKNGTCPWLRPDGKTQVTVEYYNDNGAMVPVRVHTVLISTQHDETVTNDEIATDLKEHVIKPIIPEKYLDEKTIFHLNPSGRFVIGGPHGDAGLTGRKIIIDTHGGWGAHGGGAFSGKDPTKVDRSGAYIVRQAAKSIVASGLARRCIVQVSYAIGVPEPLSVFVDTYKTGKIPDKEILEIVKENFDFRPGMITINLDLKRGGNGRFLKTAAYGHFGRDDPDFTWETIKPLKWEKPQS

> *Theobroma cacao* EOY06891.1

MNGPKRGPKRAQVKNRVTPASFLLTHRLRLGCHLLLSSIQIETQQQHVLHLRGGFINGSHSALRVTISPQIQISPDSNESSLDYASEGSRKQAAPSLILHSRIQEMETFLFTSESVNEGHPDKLCDQISDAVLDACLSQDPDSKVACETCTKTNMVMIFGEITTKANVDYEKIVRDTCRGIGFTSDDVGLDADNCKVLVNIEQQSPDIAQGVHGHLTKSPEEIGAGDQGHMFGYATDETSELMPLSHVLATKLGARLTEVRKNGTCPWLRPDGKTQVTVEYYNDKGAMVPVRVHTVLISTQHDETVTNDEIAADLKEHVIKPVIPDKYLDEKTIFHLNPSGRFVIGGPHGDAGLTGRKIIIDTYGGWGAHGGGAFSGKDPTKVDRSGAYIVRQAAKSIVANELARRCIVQVSYAIGVPEPLSVFVDTYGTGKIPDKEILKIVKENFDFRPGMISIKLDLKRGGNGRFLKTAAYGHFGRDDPDFTWEVVKPLKWDKAQA

> *Gossypium hirsutum* ADN96174.1

METFLFTSESVNEGHPDKLCDQVSDAVLDACLAQDPDSKVACETCTKTNMVMVFGEITTKANVDYEKIVRDTCRSIGFVSDDVGLDADNCKVLVNIEQQSPDIAQGVHGHFTKRPEEIGAGDQGHMFGYATDETPEFMPLSHVLATKLGARLTDVRKNGTCPWLRPDGKTQVTVEYYNDNGAMVPVRVHTVLISTQQYETVTNDEIAADLKEHVIKPVIPEKYLDEKTIFHLNPSGRFVIGGPHGDAGLTGRKIIIDTYGGWGAHGGGAFSGKDPTKVDRSGAYIVRQAAKSIVANGLARRCIVQVSYAIGVPEPLSVFVDSYGTGKIPDKEILQIVKENFDFRPGMITINLDLKRGGNSRFLKTAAYGHFGRDDPDFTWEAVKPLKWDKPQS

> *Prunus persica* GenBank: AGF95108.1

METFLFTSESVNEGHPDKLCDQISDAVLDACLAQDADSKVACETCTKTNMVMVFGEITTKANVDYEKIVRETCRNIGFISDDVGLDADNCKVLVNIEQQSPDIAQGVHGHFTKRPEEIGAGDQGHMFGYATDETPELMPLSHVLATKLGARLTEVRKNGTCPWLRPDGKTQVTVEYYNENGAMVPVRVHTVLISTQHDETVTNDEIAADLKEHVIKPVVPEKYLDEKTIFHLNPSGRFVIGGPHGDAGLTGRKIIIDTYGGWGAHGGGAFSGKDPTKVDRSGAYIVRQAAKSIVANGLARRALVQVSYAIGVPEPLSVFVDTYGTGKIPDKEILKIVKETFDFRPGMITINLDLKRGGGGRFLKTAAYGHFGRDDPDFTWEVVKPLKWEKPQS

> *Lycoris radiata* AFC88125.1

MADTFLEMADTFLFTSESVNEGHPDKLCDQISDAVLDACLAEDPDSKVVCETCTKTNMVMVFGEITTKANVDYEKIIRDTCRSIGFVSDDVGLDADNCKVLVNIEQQSPDIAQGVHGHFTKRPEEIGAGDQGHMFGYATDETPELMPLSHVLATKLGARLTEVRKNGTCPWLRPDGKTQVTIEYRNDHGAMVPLRVHTVLISTQHDETVTNDEIAADLKAHVIKPVVPEQYLDEKTIFHLNPSGRFVIGGPHGDAGLTGRKIIIDTYGGWGAHGGGAFSGKDPTKVDRSGAYIVRQAAKSIVANGLARRCIVQVSYAIGVPEPLSVFVDTYGTGKIPDKEILNIVKENCDFRPGMITINLDLKRGGNGRFLKTAAYGHFGRDDPDFTWEIVRPLKWEKPAA

> *Arabidopsis thaliana* NP_188365.1

MESFLFTSESVNEGHPDKLCDQISDAILDACLEQDPESKVACETCTKTNMVMVFGEITTKANVDYEQIVRKTCREIGFVSADVGLDADNCKVLVNIEQQSPDIAQGVHGHLTKKPEEVGAGDQGHMFGYATDETPELMPLTHVLATKLGAKLTEVRKNGTCPWLRPDGKTQVTIEYINESGAMVPVRVHTVLISTQHDETVTNDEIAADLKEHVIKPVIPEKYLDEKTIFHLNPSGRFVIGGPHGDAGLTGRKIIIDTYGGWGAHGGGAFSGKDPTKVDRSGAYIVRQAAKSIVASGLARRVIVQVSYAIGVPEPLSVFVDSYGTGKIPDKEILEIVKESFDFRPGMISINLDLKRGGNGRFLKTAAYGHFGRDDADFTWEVVKPLKSNKVQA

> *Oryza officinalis* CAJ45561.1

TFLFTSESVNEGHPDKLCDQVSDAVLDACLAEDPDSKVACETCTKTNMVMVFGEITTKANVDYEKIVRETCRNIGFVSADVGLDADHCKVLVNIEQQSPDIAQGVHGHFTKRPEEIGAGDQGHMFGYATDETPELMPLSHVLATKLGARLTEVRKNGTCAWLRPDGKTQVTVEYRNESGAMVPVRVHTVLISTQHDETVTNDEIAADLKEHVIKPVIPEQYLDEKTIFHLNPSGRFVIGGPHGDAGLTGRKIIIDTYGGWGAHGGGAFSGKDPTKVDRSGAYIARQAAKSIVASGLARRCIVQVSYAIGVPEPLSVFVDTYGTGRIPDKEILKIVKENFDFRPGMIIINLDLKKGGNGRYLKTAAYGHFGRDDPDFTWEVVKPLKWEKP

> *Gladiolus grandiflorus* ADM18304.1

MAGEDTFLFTSESVNEGHPDKLCDQISDAVLDACLTQDPDSKVACETCTKTNMVMVFGEITTKANIDYEKIVRDTCRSIGFVSADVGLDADRCKVLVNIEQQSPDIAQGVHGHFTKKPEEIGAGDQGHMFGYATDETPELMPLSHVLATKLGAKLTEVRKNGTCAWLRPDGKTQVTVEYRNDNGAMVPLRVHTILISTQHDETVTNDEIAADLKEHVIKPVVPLRYLDDKTIFHLNPSGRFVIGGPHGDAGLTGRKIIIDTYGGWGAHGGGAFSGKDPTKVDRSGAYIVRQAAKSIVASGMARRCIVQVSYAIGVPEPLSVFVDSYGTGRIPDKEILKIVKESFDFRPGMITINLDLKRGGNGRFLKTAAYGHFGRDDADFTWETVKPLKWEKPSVA
