# Supplementary material for: Molecular Cloning, Characterization and Expression Analysis of the SAMS Gene during Adventitious Root Development in IBA-Induced Tetraploid Black Locust
Source: PLoS One. 2014 Oct 6;9(10):e108709. doi: 10.1371/journal.pone.0108709 (PMC4186884; doi:10.1371/journal.pone.0108709)
Supplement: Materials S4 — The raw data of Figure 7 . Raw data refering to relative expression levels of TrbSAMS (A), TrbSAMDC (B), TrbPAO (C), and TrbACS (D) during the different IBA-induced and untreated different rooting phases in softwood cuttings of tetraploid black locust. SD = Standard Deviation, n = 3. (DOC) [file pone.0108709.s005.doc]

**Supplementary material 5: raw data of Figure 7**

**Raw data refering to relative expression levels of *TrbSAMS* (A), *TrbSAMDC* (B), *TrbPAO* (C), and *TrbACS* (D) during the different IBA-induced** **and untreated different rooting phases in softwood cuttings of tetraploid black locust.**

| *TrbSAMS* |  |  |  |  |
| --- | --- | --- | --- | --- |
|  | CK | SD | IBA | SD |
| I | 0.90 | 0.11 | 0.90 | 0.11 |
| C | 3.91 | 0.09 | 3.31 | 0.16 |
| RP | 0.84 | 0.12 | 3.31 | 0.45 |
| AR | 0.60 | 0.15 | 3.35 | 0.22 |
|  |  |  |  |  |
| *TrbSAMDC* |  |  |  |  |
|  | CK | SD | IBA | SD |
| I | 1.00 | 0.11 | 1.00 | 0.11 |
| C | 2.16 | 0.40 | 0.83 | 0.06 |
| RP | 0.91 | 0.09 | 2.57 | 0.22 |
| AR | 0.20 | 0.11 | 1.27 | 0.34 |
|  |  |  |  |  |
| *TrbPAO* |  |  |  |  |
|  | CK | SD | IBA | SD |
| I | 0.71 | 0.11 | 0.71 | 0.11 |
| C | 3.14 | 0.38 | 1.22 | 0.10 |
| RP | 0.73 | 0.23 | 3.56 | 0.52 |
| AR | 0.33 | 0.06 | 1.86 | 0.52 |
|  |  |  |  |  |
| *TrbACS* |  |  |  |  |
|  | CK | SD | IBA | SD |
| I | 0.81 | 0.11 | 0.81 | 0.11 |
| C | 0.70 | 0.16 | 1.21 | 0.18 |
| RP | 1.72 | 0.06 | 1.23 | 0.27 |
| AR | 2.72 | 0.15 | 25.19 | 0.27 |
