# Supplementary material for: Molecular Cloning, Characterization and Expression Analysis of the SAMS Gene during Adventitious Root Development in IBA-Induced Tetraploid Black Locust
Source: PLoS One. 2014 Oct 6;9(10):e108709. doi: 10.1371/journal.pone.0108709 (PMC4186884; doi:10.1371/journal.pone.0108709)
Supplement: Materials S5 — The raw data of Figure 8 . Raw data refering to expression of of TrbSAMS, TrbSAMDC, TrbPAO, and TrbACS in shoot, leaf, bark, and root. SD = Standard Deviation, n = 3. (DOC) [file pone.0108709.s006.doc]

**Supplementary material 6: raw data of Figure 8**

**Raw data refering to expression of of *TrbSAMS*, *TrbSAMDC*, *TrbPAO*, and *TrbACS* in shoot, leaf, bark, and root.**

| gene | Shoot | SD | Leaf | SD | Bark | SD | Root | SD |
| --- | --- | --- | --- | --- | --- | --- | --- | --- |
| *TrbSAMS* | 0.59 | 0.03 | 1.44 | 0.06 | 3.35 | 0.22 | 0.36 | 0.23 |
| *TrbSAMDC* | 0.28 | 0.07 | 0.73 | 0.08 | 1.27 | 0.34 | 0.55 | 0.12 |
| *TrbPAO* | 0.40 | 0.14 | 1.14 | 0.10 | 1.86 | 0.52 | 0.61 | 0.07 |
| *TrbACS* | 0.60 | 0.04 | 4.00 | 0.11 | 25.19 | 0.27 | 7.62 | 0.09 |
