# Supplementary material for: Molecular Cloning, Characterization and Expression Analysis of the SAMS Gene during Adventitious Root Development in IBA-Induced Tetraploid Black Locust
Source: PLoS One. 2014 Oct 6;9(10):e108709. doi: 10.1371/journal.pone.0108709 (PMC4186884; doi:10.1371/journal.pone.0108709)
Supplement: Materials S6 — The raw data of Figure 9 . Raw data of SAMS activity (A), polymine content (B), ACS activity (C), and ethylene production (D) during the different IBA-induced and untreated different rooting phase of softwood cuttings in tetraploid black locust. SD = Standard Deviation, n = 3. (DOC) [file pone.0108709.s007.doc]

**Supplementary material 7: raw data of Figure 9**

| Ethylene production | | |  |  |
| --- | --- | --- | --- | --- |
|  | CK | SD | IBA | SD |
| I | 23.56 | 1.64 | 23.56 | 1.74 |
| C | 32.87 | 1.63 | 35.74 | 1.63 |
| RP | 37.54 | 1.54 | 48.88 | 1.54 |
| AR | 65.44 | 1.55 | 89.48 | 1.55 |
| ACS activity | |  |  |  |
| CK | | SD | IBA | SD |
| I | 43.56 | 1.54 | 43.56 | 1.74 |
| C | 62.87 | 1.63 | 75.74 | 1.63 |
| RP | 87.54 | 1.54 | 88.88 | 1.54 |
| AR | 105.44 | 1.65 | 119.48 | 1.85 |
| SAMS activity | |  |  |  |
| CK | | SD | IBA | SD |
| I | 63.56 | 1.72 | 63.56 | 1.73 |
| C | 214.87 | 1.91 | 194.74 | 2.03 |
| RP | 156.54 | 1.88 | 210.88 | 1.99 |
| AR | 78.44 | 1.79 | 190.48 | 1.91 |
| Polymines contents | | |  |  |
| CK SD | | | IBA | SD |
| I | 63.56 | 2.02 | 63.56 | 2.03 |
| C | 134.87 | 2.11 | 234.74 | 2.23 |
| RP | 156.54 | 1.98 | 298.88 | 1.99 |
| AR | 78.44 | 1.99 | 109.48 | 2.11 |

**Raw datum of SAMS activity (A), polymine content (B), ACS activity (C), and ethylene production (D) during the different IBA-induced and untreated different rooting phase of softwood cuttings in tetraploid black locust.**
